# The Efficacy and Safety of Lenalidomide plus Rituximab in an Orbital Relapse of Diffuse Large B-Cell Lymphoma

**DOI:** 10.1155/2019/2845130

**Published:** 2019-09-12

**Authors:** R. Palmieri, F. Esposito, F. Meconi, V. M. Rapisarda, L. Anemona, G. Paterno, S. Vaccarini, D. Nasso, L. Pupo, M. Cantonetti

**Affiliations:** Fondazione Policlinico Tor Vergata, Dipartimento di Biomedicina e Prevenzione, Rome, Italy

## Abstract

A 74-year-old male with diffuse large B-cell lymphoma, with an Ann Arbor stage IV-A, was submitted to immune-chemotherapy in 2014, with complete remission of the disease. Two years later, he presented with a left eye swelling leading to exophthalmos and blurred vision. A core biopsy was performed and revealed a local relapse of the disease. He was considered unfit for intensive salvage chemotherapy and was treated with a combination of rituximab and lenalidomide. After six courses of rituximab plus lenalidomide, the patient showed complete remission and was submitted to maintenance therapy with lenalidomide. After 24 months since the start of lenalidomide monotherapy, we did not observe any progression. In this experience, rituximab plus lenalidomide, without radiotherapy, was a safe and effective therapeutic combination in an elderly patient with a localized relapse of DLBCL who was unfit to receive more aggressive therapies.

## 1. Background

Diffuse large B-cell lymphoma (DLBCL), with an annual incidence of 7-8 cases per 100,000 people per year, is the most common subtype of aggressive non-Hodgkin's lymphoma [1], and notwithstanding recent chemotherapeutic advances, disease relapse occurs in up to half of all patients [2].

The extranodal presentation to the head at the onset of the disease is very uncommon [3]. However, orbital lymphomas represent about 5–15% of extranodal lymphomas and approximately 50% of all primary malignant tumors of the orbit. The incidence of an isolated recurrence at the orbit remains unknown [4]. It usually occurs in elderly patients. It is characterized by a poor prognosis, and until now, it lacks standard therapy [5].

Multiple therapies targeting the biological pathways of B-cell lymphomas are under clinical evaluation. Among them, lenalidomide, an immunomodulatory agent with both tumoricidal and immunomodulatory effects, appears particularly promising. Its tumoricidal effects include inhibition of vascular endothelial growth factor-mediated microvessels formation, leading to cancer cells' cycle arrest and apoptosis [6]. Immunomodulatory effects of lenalidomide include inhibition of proinflammatory cytokines such as tumor necrosis factor *α*, increased the cytotoxicity of natural killer (NK) cells, inhibition of regulatory T cells, and increased anti-inflammatory cytokines [7–9]. The association of lenalidomide with the anti-CD20 monoclonal antibody rituximab has been studied in several trials, showing encouraging results [10, 11].

## 2. Case Presentation

A 74-year-old male presented to our department with a red, ulcerated plaque on the left arm with three months of duration. Recently, the lesion was rapidly increasing in size and started bleeding. Excisional biopsy was performed, and the material was sent for histopathological examination. Microscopic examination revealed diffuse infiltrates of large noncleaved cells, with large nuclei and conspicuous nucleoli. Immunohistochemical evaluation revealed the abnormal cells to be CD20+ Bcl6+ MUM1+ CD10–c-Myc– and CD3–. The proliferative index (Ki 67) was 90%. A diagnosis of nongerminal center diffuse large B-cell non-Hodgkin's lymphoma (non-GCB DLBCL) was established. The bone marrow did not reveal any involvement of lymphoma. A fluorodeoxyglucose positron emission tomography (FDG-PET) was performed and it showed a diffuse involvement of mediastinal nodes.

The patient was subsequently started on systemic chemotherapy with rituximab combined with liposomal doxorubicin, cyclophosphamide, vincristine, and prednisone (R-COMP) for six cycles, followed by involved field radiotherapy on the arm. He well tolerated the therapy and obtained a complete remission.

**Figure 1:**
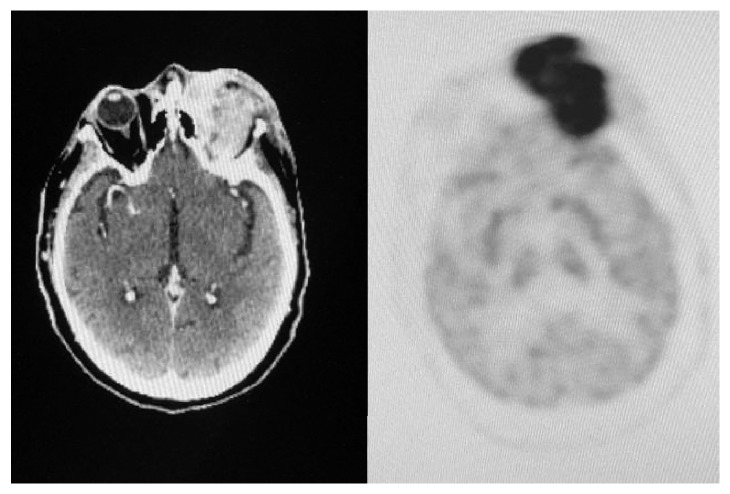
The FDG-PET/CT scan at relapse showed a huge tumor mass with high metabolic rate.

Two years after the completion of therapy, the patient came to our observation with a left eye swelling leading to exophthalmos and blurred vision (Figure 1). A core biopsy was performed, and it revealed a disease with the same immunohistochemical panel of the diagnosis. Magnetic resonance imaging (MRI) showed a high-density process involving the left orbit and the surrounding soft tissues. An FDG-PET/Tc excluded any systemic involvement.

Bone marrow biopsy was not performed due to patient refusal.

**Figure 2:**
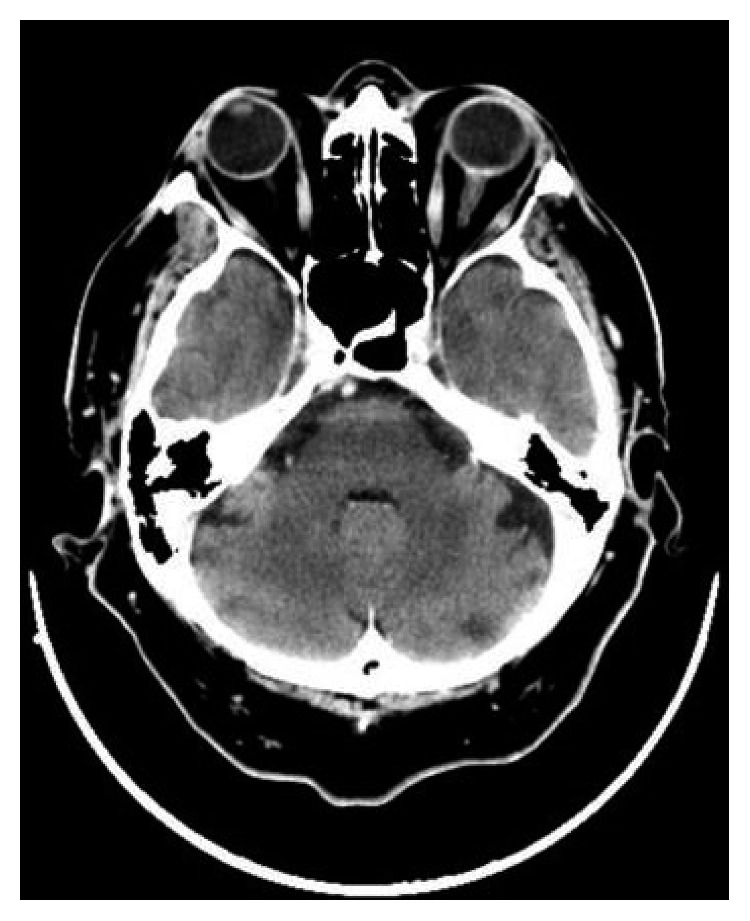
The CT scan performed after the end of the salvage therapy showed complete remission of the disease.

At the time of the relapse, the patient was 76 years old and considered ineligible for high-dose second-line chemotherapy. Moreover, radiotherapy was not considered for the large extension of the disease because of the long-term side effects of rays on the patient's sight. In the absence of standardized therapy for these patients, we chose the combination of rituximab (375 mg/mq D1) plus lenalidomide (15 mg D1–21) every 28 days for 6 courses. At the end of the therapy, complete remission was confirmed by MRI and FDG-PET/CT scan evaluation (Figure 2).

After remission, lenalidomide (15 mg D1–21, every 28 days) monotherapy maintenance was started and planned to be continued until progression or unacceptable toxicity. After 24 months since the start of lenalidomide monotherapy, the patient is still in complete remission with an excellent quality of life.

## 3. Discussion and Conclusions

Diffuse large B-cell lymphoma is the most common subtype of non-Hodgkin's lymphoma, accounting for approximately 30% of new cases. Despite the high rate of response to first-line standard chemotherapy R-CHOP, 30–40% of patients are refractory or relapse. High-dose chemotherapy followed by autologous stem cell transplant is considered a good option in relapsed/refractory young fit patients, with rates of overall survival at 4 years up to 60% [12]. Nevertheless, for patients not eligible for intensive salvage therapy, including most >70 years of age, front-line represents almost always the only chance for a cure, especially in non-GCB subtypes [13].

In our case, a 77-year-old patient presented with a relapsed non-GCB DLCBL involving the orbit. Long and even short-term prognosis of this localization in elderly patients is considered unfavorable, and we do not have a recommended therapy yet. Our patient was not eligible for standard high-dose chemotherapy, and radiotherapy was considered palliative. We decided to use the association of lenalidomide and rituximab as a manageable and well-tolerated therapy [14]. The efficacy of lenalidomide monotherapy has been investigated in heavily-pretreated DLBCL, resulting in higher overall response rate and longer progression-free survival as compared to standard intensive chemotherapy [15].

Furthermore, as recently observed in large multicenter randomized trials, a positive trend favoring lenalidomide plus R-CHOP versus R-CHOP alone was observed in previously untreated high-risk DLBCL [16]. Moreover, this drug association has also been tested in refractory/relapsed aggressive NHL involving the central nervous system and the ocular district, recording promising results [17]. Of note, the incidence of grade 3-4 toxicities was found remarkably higher among patients treated with standard chemotherapy as compared to those treated with lenalidomide plus rituximab [18], encouraging the use of this drug association for patients considered unsuitable for intensive treatment.

Finally, lenalidomide as maintenance therapy, meaningfully improved the outcome of patients with DLBCL if compared to placebo. Intriguing, the progression-free survival benefit of lenalidomide maintenance was equally important in patients who achieved complete remission as in those achieving a partial remission [19].

Therefore, our case report shows that the association of lenalidomide plus rituximab could be an option in the treatment of isolated orbital relapse of systemic non-GCB DLBCL. This strategy, improved by the following lenalidomide maintenance treatment, might be an attractive and well-tolerated therapeutic option in relapsed/refractory elderly patients who are unfit for intensive salvage chemotherapy.

## References

[1] Morton L. M., Wang S. S., Devesa S. S., Hartge P., Weisenburger D. D., Linet M. S. (2006). Lymphoma incidence patterns by WHO subtype in the United States, 1992–2001. *Blood*.

[2] Feugier P., van Hoof A., Sebban C. (2005). Long-term results of the R-CHOP study in the treatment of elderly patients with diffuse large B-cell lymphoma: a study by the groupe d’etude des lymphomes de l’adulte. *Journal of Clinical Oncology*.

[3] Newton R., Ferlay J., Beral V., Devesa S. S. (1997). The epidemiology of non-Hodgkin’s lymphoma: comparison of nodal and extra-nodal sites. *International Journal of Cancer*.

[4] Margo C. E., Mulla Z. D. (1998). Malignant tumors of the orbit. *Ophthalmology*.

[5] Stacy R. C., Jakobiec F. A., Herwig M. C., Schoenfield L., Singh A., Grossniklaus H. E. (2012). Diffuse large B-cell lymphoma of the orbit: clinicopathologic, immunohistochemical, and prognostic features of 20 cases. *American Journal of Ophthalmology*.

[6] Lu L., Payvandi F., Wu L. (2009). The anti-cancer drug lenalidomide inhibits angiogenesis and metastasis via multiple inhibitory effects on endothelial cell function in normoxic and hypoxic conditions. *Microvascular Research*.

[7] Corral L. G., Haslett P. A., Muller G. W. (1999). Differential cytokine modulation and T cell activation by two distinct classes of thalidomide analogues that are potent inhibitors of TNF-alpha. *Journal of Immunology*.

[8] Galustian C., Meyer B., Labarthe M.-C. (2009). The anti-cancer agents lenalidomide and pomalidomide inhibit the proliferation and function of T regulatory cells. *Cancer Immunology, Immunotherapy*.

[9] Hsu A. K., Quach H., Tai T. (2011). The immunostimulatory effect of lenalidomide on NK-cell function is profoundly inhibited by concurrent dexamethasone therapy. *Blood*.

[10] Chiappella A., Tucci A., Castellino A. (2013). Lenalidomide plus cyclophosphamide, doxorubicin, vincristine, prednisone and rituximab is safe and effective in untreated, elderly patients with diffuse large B-cell lymphoma: a phase I study by the Fondazione Italiana Linfomi. *Haematologica*.

[11] Nowakowski G. S., LaPlant B., Macon W. R. (2015). Lenalidomide combined with R-CHOP overcomes negative prognostic impact of non-germinal center B-cell phenotype in newly diagnosed diffuse large B-cell lymphoma: a phase II study. *Journal of Clinical Oncology*.

[12] Robinson S. P., Boumendil A., Finel H. (2016). Autologous stem cell transplantation for relapsed/refractory diffuse large B-cell lymphoma: efficacy in the rituximab era and comparison to first allogeneic transplants. A report from the EBMT lymphoma working party. *Bone Marrow Transplantation*.

[13] Morrison V. A., Hamlin P., Soubeyran P. (2015). Approach to therapy of diffuse large B-cell lymphoma in the elderly: the international society of geriatric oncology (SIOG) expert position commentary. *Annals of Oncology*.

[14] Zinzani P. L., Pellegrini C., Gandolfi L. (2011). Combination of lenalidomide and rituximab in elderly patients with relapsed or refractory diffuse large B-cell lymphoma: a phase 2 trial. *Clinical Lymphoma Myeloma and Leukemia*.

[15] Czuczman M. S., Trněný M., Davies A. (2017). A phase 2/3 multicenter, randomized, open-label study to compare the efficacy and safety of lenalidomide versus investigator’s choice in patients with relapsed or refractory diffuse large B-cell lymphoma. *Clinical Cancer Research*.

[16] Vitolo U., Witzig T. E., Gascoyne R. D. (2019). ROBUST: first report of phase III randomized study of lenalidomide/R-CHOP (R^2^-CHOP) vs placebo/R-CHOP in previously untreated ABC-type diffuse large B-cell lymphoma. *Hematological Oncology*.

[17] Ghesquieres H., Chevrier M., Laadhari M. (2019). Lenalidomide in combination with intravenous rituximab (REVRI) in relapsed/refractory primary CNS lymphoma or primary intraocular lymphoma: a multicenter prospective “proof of concept” phase II study of the French oculo-cerebral lymphoma (LOC) network and the lymphoma study association (LYSA). *Annals of Oncology*.

[18] Morschhauser F., Fowler N. H., Feugier P. (2018). Rituximab plus lenalidomide in advanced untreated follicular lymphoma. *New England Journal of Medicine*.

[19] Thieblemont C., Tilly H., Gomes da Silva M. (2017). Lenalidomide maintenance compared with placebo in responding elderly patients with diffuse large B-cell lymphoma treated with first-line rituximab plus cyclophosphamide, doxorubicin, vincristine, and prednisone. *Journal of Clinical Oncology*.

